# Physical multimorbidity and psychosis: comprehensive cross sectional analysis including 242,952 people across 48 low- and middle-income countries

**DOI:** 10.1186/s12916-016-0734-z

**Published:** 2016-11-22

**Authors:** Brendon Stubbs, Ai Koyanagi, Nicola Veronese, Davy Vancampfort, Marco Solmi, Fiona Gaughran, André F. Carvalho, John Lally, Alex J. Mitchell, James Mugisha, Christoph U. Correll

**Affiliations:** 1 Physiotherapy Department, South London and Maudsley NHS Foundation Trust, Denmark Hill, London, SE5 8AZ UK; 2 Health Service and Population Research Department, Institute of Psychiatry, Psychology and Neuroscience, King’s College London, De Crespigny Park, London, Box SE5 8AF, UK; 3 Research and Development Unit, Parc Sanitari Sant Joan de Déu, Universitat de Barcelona, Fundació Sant Joan de Déu, Dr. Antoni Pujadas, 42, Sant Boi de Llobregat, Barcelona, 08830 Spain; 4 Instituto de Salud Carlos III, Centro de Investigación Biomédica en Red de Salud Mental, CIBERSAM, Monforte de Lemos 3-5 Pabellón 11, Madrid, 28029 Spain; 5 Geriatrics Division, Department of Medicine-DIMED, University of Padova, Padova, Italy; 6 Institute of Clinical Research and Education in Medicine (IREM), Padova, Italy; 7 KU Leuven Department of Rehabilitation Sciences, Leuven, Belgium; 8 KU Leuven, University Psychiatric Center KU Leuven, Leuven-Kortenberg, Belgium; 9 Department of Neurosciences, University of Padova, Padova, Italy; 10 Local Health Unit ULSS 17, Mental Health Department, Monselice, Padova, Italy; 11 Department of Psychosis Studies, Institute of Psychiatry, Psychology and Neuroscience King’s College London, London, UK; 12 Department of Psychiatry and Translational Psychiatry Research Group, Faculty of Medicine, Federal University of Ceará, Fortaleza, CE Brazil; 13 Department of Psychiatry, Royal College of Surgeons in Ireland, Dublin, Ireland; 14 Department of Cancer and Molecular Medicine, University of Leicester, Leicester, UK; 15 Kyambogo University, Kampala, Uganda; 16 Butabika National Referral and Mental Health Hospital, Kampala, Uganda; 17 The Zucker Hillside Hospital, Psychiatry Research, Northwell Health, Glen Oaks, New York, USA; 18 Hofstra Northwell School of Medicine, Hempstead, New York, USA

**Keywords:** Psychosis, Physical health, Multimorbidity, Psychotic experiences, Metabolism

## Abstract

**Background:**

In people with psychosis, physical comorbidities, including cardiovascular and metabolic diseases, are highly prevalent and leading contributors to the premature mortality encountered. However, little is known about physical health multimorbidity in this population or in people with subclinical psychosis and in low- and middle-income countries (LMICs). This study explores physical health multimorbidity patterns among people with psychosis or subclinical psychosis.

**Methods:**

Overall, data from 242,952 individuals from 48 LMICs, recruited via the World Health Survey, were included in this cross-sectional study. Participants were subdivided into those (1) with a lifetime diagnosis of psychosis (“psychosis”); (2) with more than one psychotic symptom in the past 12 months, but no lifetime diagnosis of psychosis (“subclinical psychosis”); and (3) without psychotic symptoms in the past 12 months or a lifetime diagnosis of psychosis (“controls”). Nine operationalized somatic disorders were examined: arthritis, angina pectoris, asthma, diabetes, chronic back pain, visual impairment, hearing problems, edentulism, and tuberculosis. The association between psychosis and multimorbidity was assessed by multivariable logistic regression analysis.

**Results:**

The prevalence of multimorbidity (i.e., two or more physical health conditions) was: controls = 11.4% (95% CI, 11.0–11.8%); subclinical psychosis = 21.8% (95% CI, 20.6–23.0%), and psychosis = 36.0% (95% CI, 32.1–40.2%) (*P* < 0.0001). After adjustment for age, sex, education, country-wise wealth, and country, subclinical psychosis and psychosis were associated with 2.20 (95% CI, 2.02–2.39) and 4.05 (95% CI, 3.25–5.04) times higher odds for multimorbidity. Moreover, multimorbidity was increased in subclinical and established psychosis in all age ranges (18–44, 45–64, ≥ 65 years). However, multimorbidity was most evident in younger age groups, with people aged 18–44 years with psychosis at greatest odds of physical health multimorbidity (OR = 4.68; 95% CI, 3.46–6.32).

**Conclusions:**

This large multinational study demonstrates that physical health multimorbidity is increased across the psychosis-spectrum. Most notably, the association between multimorbidity and psychosis was stronger among younger adults, thus adding further impetus to the calls for the early intervention efforts to prevent the burden of physical health comorbidity at later stages. Urgent public health interventions are necessary not only for those with a psychosis diagnosis, but also for subclinical psychosis to address this considerable public health problem.

## Background

There is now established evidence that people with psychosis experience a premature mortality gap of up to 15 years before members of the general population [1, 2]. The overwhelming majority of premature mortality cases is due to physical health comorbidities, such as cardiovascular, metabolic, and respiratory diseases [3]. Recent studies have demonstrated that people with psychosis are at greatly increased risk of cardiovascular disease [4], metabolic syndrome [5], diabetes [6], and respiratory disease [7], and that these risks are especially increased compared to similarly aged healthy populations [8, 9]. In addition, people with psychosis may be more likely to experience other physical health conditions, such as arthritis, chronic back pain [10], or edentulism, which may not increase the risk of premature mortality, but which can negatively impact quality of life, for example, via pain or social stigmatization [11].

Despite the knowledge of the heightened risk of various physical comorbidities, very little is known about multimorbidity (i.e., two or more physical health comorbidities) in people with psychosis. However, multimorbidity is an important risk concept, as it has been associated with functional decline [12], worse quality of life [13], increased risk of premature mortality [14, 15], and increased healthcare costs [16]. In the largest study to date, people with schizophrenia in Scotland (*n* = 9677) were significantly more likely to have two physical health comorbidities (OR = 1.37, 95% CI, 1.29–1.44) and three or more physical health comorbidities (OR = 1.19, 95% CI, 1.12–1.27) than general population controls [17]. Whilst helpful, the focus in this study was on primary care records limited to one country, and therefore the results are not easily generalizable. Moreover, there is increasing recognition that psychosis lies on a continuum, and that those with psychotic symptoms without a full diagnosis (subclinical psychosis) also experience an increased risk of various physical comorbidities [18–20] and mortality [21]. To our knowledge, no study has investigated multimorbidity in those with subclinical psychosis. Understanding multimorbidity patterns can help identify higher risk subgroups, which would preferentially benefit from tailored preventative and therapeutic strategies [22, 23].

Furthermore, data regarding multimorbidity in low- and middle-income countries (LMICs) are scarce and there is a lack of data on multimorbidity among those with psychosis in this setting. This lacking literature represents an important research gap since increasing trends of multimorbidity in LMICs will have considerable financial implications over the next few decades [24]. In addition, multimorbidity patterns in people with psychosis in the context of LMICs may differ from those in high-income countries. For example, the risk for cardio-metabolic diseases may differ due to limited availability of second-generation antipsychotics, many of which are known to especially increase the risk for metabolic abnormalities [6, 7]. When antipsychotics are available in LMICs, first-generation antipsychotics are commonly prescribed [25]. Particularly high-potency first generation-antipsychotics appear to have a lower cardiometabolic risk profile than most of the second-generation antipsychotics used in high-income countries [26–28]. In addition, although smoking is known to be a major risk factor for cardiovascular diseases for patients with schizophrenia in developed countries, the rate of smoking among those with schizophrenia is not elevated compared to the general population in some LMICs, where smoking rates have remained high in the general population [29].

Given these aforementioned gaps in the literature, we set out to assess the prevalence of physical comorbidity and multimorbidity patterns in adults with psychosis and sub-clinical psychosis across 48 LMICs. We hypothesized that a clinically significant proportion of people would be affected by physical multimorbidity and that there would be a dose–response relationship with psychosis, such that adults with subclinical psychosis would be in between the general population and those with established psychotic disorders.

## Methods

The World Health Survey (WHS) was a cross-sectional study undertaken in 2002–2004 in 70 countries worldwide. The aim of the study was to provide global comparable population data on health and well-being among adults. Details of the survey have been provided on the World Health Organization (WHO) website (http://www.who.int/healthinfo/survey/en/) and in previous publications [30, 31]. Briefly, single-stage random sampling and stratified multi-stage random cluster sampling were conducted in 10 and 60 countries, respectively. Stratification was conducted by sex, age, and residential area (rural/urban). Enumeration areas and households were also used as stratification units in the majority of countries. Persons aged 18 years or older with a valid home address were eligible to participate. Each member of the household had equal probability of being selected with the use of Kish tables. The data were collected in all countries using the same standardized questionnaire with some countries using a shorter version (mainly high-income countries). Since the questions on psychosis were not included in the shorter version, this meant that information on psychosis was absent from the vast majority of high-income countries. The questionnaire was translated into multiple languages and was back- and forward-translated using a standard WHO protocol, and checked by linguists to ensure comparability. Data collection was conducted either by face-to-face interviews or via telephone (Luxembourg and Israel) by trained interviewers who had at least a high school-level education. The interviewers attended a week-long course and conducted practice field interviews prior to the actual survey. The individual response rate across all countries was 98.5% [32]. Ethical approval to conduct this survey was obtained from ethical boards at each study site. Sampling weights were generated to adjust for non-response and the population distribution reported by the United Nations Statistical Division. Informed consent was obtained from all participants.

The current study used data from 69 countries that have been made publically available. The data were nationally representative in all countries with the exception of China, Comoros, the Republic of Congo, Ivory Coast, India, and Russia. Countries without any sampling information (10 countries – Austria, Belgium, Denmark, Germany, Greece, Guatemala, Italy, Netherlands, Slovenia, and UK) were not included in the analysis. Of the remaining 59 countries, nine (Finland, France, Ireland, Israel, Luxembourg, Norway, Portugal, Sweden, and Turkey) were subsequently excluded due to missing information on psychosis. Furthermore, the two remaining high-income countries (Spain and United Arab Emirates) were also excluded. Thus, the final sample included 48 countries (21 low-income and 27 middle-income countries based on the World Bank classification in 2003).

### Physical health conditions

A total of nine physical conditions were assessed, representing all physical conditions available in the WHS. Arthritis, asthma, and diabetes mellitus were based on self-reported lifetime diagnosis. For angina pectoris, in addition to a self-reported diagnosis, a symptom-based diagnosis based on the Rose questionnaire was also used [33]. Chronic back pain was defined as having had back pain (including disc problems) everyday during the last 30 days. Visual impairment was defined as having extreme difficulty in seeing and recognizing a person that the participant knows across the road (i.e., from a distance about 20 meters) [34]. A validity study showed that this response likely corresponds to WHO definitions of visual impairment (20/60 or 0.48 logMAR) [34]. The participant was considered to have hearing problems if the interviewer observed this condition at the end of the survey. Edentulism was assessed by the question “Have you lost all your natural teeth?” Those who responded affirmatively were considered to have edentulism. Finally, a tuberculosis diagnosis was based on past 12-month symptoms and was defined as (1) having had a cough that lasted for three weeks or longer; and (2) having had blood in phlegm or coughed up blood [35]. We calculated the total number of these conditions while allowing for one missing variable in order to retain a larger sample size. Multimorbidity was defined as having at least two conditions, in line with previously used definitions [24].

### Psychosis diagnosis and psychotic symptoms

Participants were asked whether they had ever received a diagnosis of schizophrenia or psychosis. All participants, regardless of a psychosis diagnosis, were asked questions on positive psychotic symptoms which came from the WHO Composite International Diagnostic Interview (CIDI) 3.0 [36]. This psychosis module has been reported to be highly consistent with clinician ratings [37]. The hallucinations question excluded conditions associated with sleep-related states or substance use. Specifically, respondents were asked the following questions with answer options ‘yes’ or ‘no’: During the last 12 months, have you experienced (1) ‘A feeling something strange and unexplainable was going on that other people would find hard to believe?’ (delusional mood); (2) ‘A feeling that people were too interested in you or there was a plot to harm you?’ (delusions of reference and persecution); (3) ‘A feeling that your thoughts were being directly interfered or controlled by another person, or your mind was being taken over by strange forces?’ (delusions of control); (4) ‘An experience of seeing visions or hearing voices that others could not see or hear when you were not half asleep, dreaming or under the influence of alcohol or drugs?’ (hallucinations).

Individuals who endorsed at least one of the four abovementioned psychotic symptoms were considered to have psychotic symptoms. Based on information on psychosis diagnosis and psychotic symptoms, a three-category psychosis variable was constructed: (1) no psychosis diagnosis and no psychotic symptoms (control group); (2) at least one psychotic symptom but no psychosis diagnosis (subclinical psychosis group); and (3) psychosis diagnosis (psychosis group).

### Other variables

Information was also examined on age, sex, country-wise wealth, and education. Principal component analysis based on 15–20 assets was conducted to establish country-wise wealth quintiles. Specifically, based on information on whether the participant owns items, such as a bicycle, refrigerator, washing machine, computer, etc., we calculated a wealth score for each individual by weighting each asset by the coefficient of the first principal component [38]. Education (highest level achieved) was categorized as no formal education, primary education, secondary or high school completed, or tertiary education completed.

### Statistical analysis

The statistical analysis was performed with Stata 14.1 (Stata Corp LP, College station, Texas). Descriptive analysis was conducted to characterize the study sample using weighted means (± standard deviations (SDs)), proportions, and unweighted Ns. The difference in sample characteristics by the presence of multimorbidity or psychosis was tested by χ^2^ tests and Student’s t-tests (or one way ANOVA) for categorical and continuous variables, respectively. Tetrachoric correlations, which assess the relationship between each pair of physical health conditions (as multimorbidity was defined as having at least two physical health conditions), were calculated for those with subclinical psychosis or a psychosis diagnosis. Multivariable logistic regression analysis was performed to assess the association between psychosis (independent variable) and multimorbidity (dependent variable), adjusting for age, sex, education, wealth, and country. Analyses stratified by country-income level (low-income or middle-income countries) and age groups (18–44, 45–64, ≥ 65 years) were also conducted. Adjustment for country was performed by including dummy variables in the models, as in previous WHS publications [32, 39]. The sample weighting and the complex study design were taken into account in the analyses. Results from the logistic regression models are presented as odds ratios (ORs) with 95% confidence intervals (CIs). The level of statistical significance was set at *P* < 0.05.

## Results

Data on 242,952 individuals from 48 countries were available for the current study. Data on psychosis and number of physical medical conditions were missing from 12.5% and 13.9%, respectively. Details on the sample characteristics are provided in Table 1. The mean (SD) age of the sample was 38.4 (16.0) years, and 50.6% were female. The prevalence of subclinical psychosis and psychosis diagnosis were 13.8% and 1.1%, respectively, while that of multimorbidity (i.e., two or more physical health conditions) was 13.2%.

**Table 1:** Characteristics of the sample (overall and by presence of multimorbidity)

			Multimorbidity			
	Total		No		Yes	
Characteristic	Unweighted N		Unweighted N		Unweighted N	
Psychosis category						
Control	179,429	85.1	160,143	86.8	19,286	74.1
Subclinical psychosis^a^	25,493	13.8	19,883	12.4	5610	22.9
Psychosis diagnosis^b^	2224	1.1	1502	0.8	722	2.9
Sex						
Male	93,358	49.4	84,268	51.2	9090	37.4
Female	115,846	50.6	98,964	48.8	16,882	62.6
Age, years (Mean (SD))		38.4 (16.0)		36.1 (14.5)		53.5 (16.8)
Education						
No formal	48,343	26.4	39,043	24.5	9300	39.2
Primary	71,356	31.4	62,449	31.5	8907	31.0
Secondary completed	72,087	32.7	66,072	34.3	6015	22.4
Tertiary completed	17,287	9.5	15,564	9.8	1723	7.4
Wealth (quintiles)						
Poorest	47,582	20.3	40,246	19.5	7336	25.5
Poorer	41,449	20.0	35,989	19.7	5460	21.9
Middle	37,705	19.8	33,350	20.0	4355	19.0
Richer	35,378	19.9	31,597	20.3	3781	17.2
Richest	33,305	19.9	30,111	20.5	3194	16.3

Data are percentages unless otherwise stated

^a^Subclinical psychosis refers to having at least one of delusional mood, delusions of reference and persecution, delusions of control, and hallucinations in the past 12 months but without a psychosis diagnosis

^b^Psychosis diagnosis refers to self-reported lifetime diagnosis of schizophrenia/psychosis

The differences in all sample characteristics between those with and without multimorbidity were statistically significant (*P* < 0.0001)

**Fig. 1:**
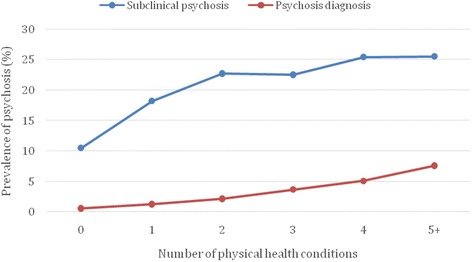
Prevalence of psychosis by number of physical health conditions. Subclinical psychosis referred to having at least one of delusional mood, delusions of reference and persecution, delusions of control, and hallucinations in the past 12 months but without a psychosis diagnosis. Psychosis diagnosis refers to self-reported lifetime diagnosis of schizophrenia/psychosis

Those with multimorbidity were significantly more likely to be older, of female sex, and lower socioeconomic status. The prevalence of physical health conditions is shown in Table 2. Overall, angina pectoris (14.9%), arthritis (13.2%), and chronic back pain (6.7%) were the most common conditions. For all conditions, there was a gradual increase in the prevalence from control, to subclinical psychosis, and to psychosis diagnosis. The prevalence of multimorbidity for control, subclinical psychosis, and psychosis diagnosis were 11.4%, 21.8%, and 36.0%, respectively (*P* < 0.0001). The prevalence of psychosis by the different frequencies of physical health conditions is illustrated in Fig. 1. A linear increase in the prevalence of subclinical psychosis and psychosis diagnosis with increasing number of comorbid physical health conditions was observed.

**Table 2:** Prevalence of physical health conditions by psychosis category

Physical health condition	Total	(a) Control	(b) Subclinical psychosis^a^	(c) Psychosis diagnosis^b^	Overall *P* value	*P* value (a) vs. (b)	*P* value (a) vs. (c)	*P* value (b) vs. (c)
Tuberculosis	1.7	1.3	3.7	7.0	<0.0001	<0.0001	<0.0001	0.0009
Visual impairment	1.3	1.2	2.0	3.8	<0.0001	<0.0001	<0.0001	<0.0001
Hearing problem	3.3	3.2	3.6	8.1	<0.0001	0.1045	<0.0001	<0.0001
Chronic back pain	6.7	5.7	11.7	15.0	<0.0001	<0.0001	<0.0001	0.0182
Edentulism	5.9	5.7	7.4	9.0	<0.0001	<0.0001	0.0011	0.1837
Arthritis	13.2	12.1	18.2	30.5	<0.0001	<0.0001	<0.0001	<0.0001
Angina pectoris	14.9	13.1	24.8	33.0	<0.0001	<0.0001	<0.0001	<0.0001
Asthma	5.1	4.5	8.5	14.8	<0.0001	<0.0001	<0.0001	<0.0001
Diabetes mellitus	3.0	2.6	5.0	6.5	<0.0001	<0.0001	<0.0001	0.0689
Number of physical health conditions								
Mean number (SD)	0.55 (0.90)	0.49 (0.86)	0.83 (0.99)	1.27 (1.33)	<0.0001	<0.0001	<0.0001	<0.0001
0	65.2	68.0	49.6	37.5	<0.0001	<0.0001	<0.0001	<0.0001
1	21.8	20.6	28.6	26.5				
2	8.6	7.5	14.1	17.8				
3	3.2	2.7	5.2	10.9				
4	1.1	0.9	1.9	5.1				
≥ 5	0.3	0.3	0.6	2.3				

Data are column percentage (i.e., the prevalence of each physical health condition among those in that psychosis category) or mean (SD). Estimates are based on weighted sample

^a^Subclinical psychosis refers to having at least one of delusional mood, delusions of reference and persecution, delusions of control, and hallucinations in the past 12 months but without a psychosis diagnosis

^b^Psychosis diagnosis refers to self-reported lifetime diagnosis of schizophrenia/psychosis

A significant positive correlation was observed for the majority of the pairs of physical health conditions among those with subclinical psychosis or psychosis diagnosis (Table 3). For those with subclinical psychosis, the strongest associations were observed for hearing problems co-occurring with visual impairment or edentulism and for arthritis co-existing with chronic back pain or angina. For those with a psychosis diagnosis, the strongest associations were observed for hearing problems co-occurring with visual impairment or edentulism, asthma co-existing with diabetes or angina or tuberculosis, and arthritis co-existing with angina. The association between psychosis and multimorbidity assessed by multivariable logistic regression is presented in Table 4. Overall, subclinical psychosis and psychosis diagnosis were associated with 2.20 (95% CI, 2.02–2.39) and 4.05 (95% CI, 3.25–5.04) times higher odds for multimorbidity. Associations in low-income and middle-income countries were similar. However, this association was stronger in the youngest age group (e.g., psychosis 18–44 years; OR = 4.68; 95% CI, 3.46–6.32) with gradual decreases in the odds being observed in the older age groups (i.e., 45–64 years and ≥ 65 years).

**Table 3:** Tetrachoric correlations of physical health conditions in subclinical psychosis and psychosis diagnosis

	Tuberculosis	Visual impairment	Hearing problem	Chronic back pain	Edentulism	Arthritis	Angina	Asthma	Diabetes
Subclinical psychosis									
Tuberculosis	1								
Visual impairment	0.0343	1							
Hearing problem	0.1030*	0.3313*	1						
Chronic back pain	0.1469*	0.2638*	0.1986*	1					
Edentulism	0.0061	0.2515*	0.3388*	0.1524*	1				
Arthritis	0.1808*	0.2033*	0.2685*	0.3180*	0.2017*	1			
Angina pectoris	0.2118*	0.1573*	0.1488*	0.2290*	0.1204*	0.3190*	1		
Asthma	0.2126*	0.1025*	0.1133*	0.1021*	0.1168*	0.1460*	0.2270*	1	
Diabetes mellitus	0.0773*	0.1850*	0.1422*	0.1224*	0.2823*	0.2222*	0.1966*	0.1709*	1
Psychosis diagnosis									
Tuberculosis	1								
Visual impairment	0.156	1							
Hearing problem	0.1201	0.3096*	1						
Chronic back pain	0.1053	0.098	0.2158*	1					
Edentulism	0.0546	0.114	0.3460*	0.0692	1				
Arthritis	0.1855*	0.2364*	0.2079*	0.2897*	0.1464*	1			
Angina pectoris	0.2844*	0.1076	0.1277*	0.1586*	0.1922*	0.3748*	1		
Asthma	0.3908*	0.1904*	0.1224	0.1290*	−0.0394	0.2360*	0.3210*	1	
Diabetes mellitus	0.2576*	0.2472*	0.1418	0.0826	0.1362	0.2136*	0.2788*	0.3674*	1

*P < 0.05

**Table 4:** Association between psychosis and multimorbidity (outcome) estimated by multivariable logistic regression analysis

	Total		Low-income countries		Middle-income countries		Age 18–44 years		Age 45–64 years		Age ≥ 65 years	
Characteristic	OR	95% CI	OR	95% CI	OR	95% CI	OR	95% CI	OR	95% CI	OR	95% CI
Psychosis category												
Control	1.00		1.00		1.00		1.00		1.00		1.00	
Subclinical psychosis^a^	2.20***	(2.02–2.39)	2.03***	(1.80–2.28)	2.43***	(2.16–2.74)	2.71***	(2.40–3.05)	1.93***	(1.68–2.20)	1.69***	(1.40–2.04)
Psychosis diagnosis^b^	4.05***	(3.25–5.04)	3.99***	(3.00–5.31)	4.07***	(2.93–5.65)	4.68***	(3.46–6.32)	3.78***	(2.77–5.16)	2.22**	(1.34–3.68)
Sex												
Male	1.00		1.00		1.00		1.00		1.00		1.00	
Female	1.66***	(1.56–1.77)	1.59***	(1.45–1.74)	1.74***	(1.59–1.89)	1.70***	(1.55–1.88)	1.77***	(1.60–1.96)	1.39***	(1.21–1.59)
Age, years	1.07***	(1.06–1.07)	1.06***	(1.06–1.06)	1.07***	(1.07–1.08)	1.07***	(1.06–1.07)	1.07***	(1.06–1.08)	1.06***	(1.05–1.07)
Education												
No formal	1.00		1.00		1.00		1.00		1.00		1.00	
Primary	0.97	(0.88–1.07)	0.99	(0.88–1.12)	0.85*	(0.74–0.99)	0.98	(0.85–1.13)	0.95	(0.82–1.11)	0.81*	(0.67–0.99)
Secondary completed	0.72***	(0.64–0.82)	0.71***	(0.60–0.85)	0.68***	(0.57–0.82)	0.74***	(0.62–0.88)	0.64***	(0.52–0.79)	0.76*	(0.58–0.99)
Tertiary completed	0.61***	(0.51–0.74)	0.54**	(0.35–0.83)	0.61***	(0.50–0.74)	0.62*	(0.43–0.90)	0.59***	(0.46–0.75)	0.66*	(0.48–0.91)
Wealth (quintiles)												
Poorest	1.00		1.00		1.00		1.00		1.00		1.00	
Poorer	0.92	(0.83–1.01)	0.94	(0.81–1.07)	0.90	(0.80–1.02)	0.91	(0.79–1.05)	0.97	(0.84–1.12)	0.82*	(0.68–1.00)
Middle	0.83***	(0.76–0.92)	0.82**	(0.71–0.93)	0.87*	(0.76–0.99)	0.79**	(0.69–0.91)	0.86*	(0.73–1.00)	0.92	(0.75–1.13)
Richer	0.84***	(0.76–0.92)	0.85*	(0.74–0.98)	0.84**	(0.74–0.96)	0.80**	(0.69–0.92)	0.90	(0.78–1.05)	0.89	(0.72–1.09)
Richest	0.85**	(0.76–0.96)	0.90	(0.77–1.05)	0.83*	(0.70–0.98)	0.79*	(0.67–0.95)	0.97	(0.81–1.15)	0.88	(0.69–1.12)

*OR* odds ratio, *CI* confidence interval

Models are adjusted for all covariates in the respective columns and country

^a^Subclinical psychosis refers to having at least one of delusional mood, delusions of reference and persecution, delusions of control, and hallucinations in the past 12 months but without a psychosis diagnosis

^b^Psychosis diagnosis refers to self-reported lifetime diagnosis of schizophrenia/psychosis

*P < 0.05, **P < 0.01, ***P < 0.001

## Discussion

Physical health comorbidities are a major problem for people suffering from psychotic disorders [7, 22, 40, 41]. However, to date, multimorbidity in subclinical or clinical psychosis has received little attention. To the best of our knowledge, the current study presents the first multinational community-based data investigating the association of psychosis and subclinical psychosis with physical health multimorbidity. The main findings of this study are that both subclinical psychosis and, even more so, a diagnosis of psychosis were associated with an increased odds for physical health multimorbidity. Specifically, subclinical psychosis was associated with a two-fold risk of multimorbidity and those with a diagnosis of psychosis were at a four times increased risk compared to those without any psychosis. Moreover, we observed a linear increase in the prevalence of subclinical psychosis and psychosis diagnosis with an increasing number of medical conditions, suggesting a close association between the number of diseases and psychosis/subclinical psychosis. The increased multimorbidity risk was evident in both low-income and middle-income countries. Of particular concern, the association between psychosis and multimorbidity was strongest in the younger age populations. Given that physical health multimorbidity can greatly increase the risk of mortality [15] and healthcare costs [16], our results confirm the considerable health strain evident among the psychosis spectrum, underscoring the fact that early intervention and prevention of both psychosis and physical health morbidities are key [22, 28, 42].

Interestingly, whilst our data found that all age ranges of people with psychosis and subclinical psychosis are at increased odds of experiencing physical health multimorbidity, people aged between 18–44 were at particular risk. Antipsychotic use, smoking and sedentary lifestyle have been suggested to be the main contributors to the increased risk of cardiometabolic diseases in schizophrenia [5, 43–45]. More recently, there has been increasing concern about the poor dietary intake and, in particular, excess consumption of saturated fats, sugar, and low fiber intake, which can increase the risk of cardiometabolic disease [46]. The increased risk for diseases such as angina pectoris and diabetes observed in subclinical psychosis may also be associated with similar risk factors seen in schizophrenia since smoking and eating problems are also highly prevalent in subclinical psychosis [29, 47], although it is unlikely that antipsychotics are commonly prescribed in this condition. Smoking, in particular, may increase the risk for multiple physical comorbidities in psychosis as it has also been associated with non-cardiometabolic diseases such as tuberculosis, edentulism, and asthma [48–50]. Finally, psychological distress arising from multiple physical health conditions may also be implicated in the increased risk for subclinical psychosis [51]. The finding that the association between multimorbidity and psychosis was stronger among the younger adds further impetus to the calls for the early intervention efforts to prevent the burden of physical health comorbidity at later stages [52].

Across the entire sample of people from LMICs, we found evidence that better education status and wealth were both protective factors for physical multimorbidity. Literature in the general population has similarly found that wealth and higher education are protective factors for better health outcomes, including mortality and physical multimorbidity [53]. The reason for this protective relationship might be an increased awareness of health risks in higher educated people, while those with a better socioeconomic status have better health coverage or greater means to access and obtain healthcare than those with less means, in particular in LMICs [54].

This study demonstrates that physical multimorbidity is a very real problem across the psychosis spectrum in LMICs. Strategies to deal with this important issue are urgently needed, particularly targeting the earlier stage of psychotic illness. For example, relevant steps towards adequately addressing multimorbidity that have been underlined before include integrating physical multimorbidity into clinical guidelines; routinely providing self-care management strategies, including advice on a healthy and active lifestyle; prioritizing the prevention of chronic conditions; and avoiding fragmented care [22, 40, 41, 53, 55]. Existing healthcare models need to be adapted to the increasing multimorbidity rates, which will need to include coordination with physical healthcare providers due to increasingly complex presentations. The adaptation of existing healthcare systems is especially relevant in LMICs, where all levels of care must be carefully planned in the context of economic restraints. The Innovative Care for Chronic Conditions framework developed by WHO provides an initial roadmap to cope with chronic conditions in developing countries, but there still is a need to fully incorporate physical multimorbidity within primary and mental healthcare settings [56]. First of all, there is a clear need to increase awareness of the importance of physical health needs of people with psychosis among primary and mental healthcare providers in LMICs. Continued medical education (a common practice in LMICs [57]) should be used to inform health providers about the importance of assessing physical health risks in people with psychosis. Health providers in LMICs need to be informed that their roles extend beyond taking care of the mental health of their patients and assume responsibility for both the mental and physical health of their patients [57]. There is also the need for mental health education institutions that train medical personnel to include physical health screening/monitoring as part of their curriculum. Policymakers should be made aware that investment in continued medical education and in the screening for physical health risks is likely to optimize mental and physical health outcomes. However, effective monitoring of metabolic risks is not sufficient on its own, as appropriate treatment is also mandatory [5, 58]. Finally, evidence indicates that the treatment of somatic diseases is often neglected in populations with psychotic disorders compared to non-psychotic healthcare users [40, 41]. For example, routine medical appointments are scheduled less often and medications for cardio-metabolic conditions [59–63] are sub-optimally prescribed when patients with psychosis are compared to those who are non-psychotic.

### Limitations

The current findings should be interpreted in light of some study limitations. First, the study was cross-sectional, therefore cause and effect cannot be deduced with certainty. Nonetheless, regardless of directionality, this survey provides a clear public health message. In addition, the diagnosis of psychosis was not assessed by a clinical interview. Moreover, the self-report measure may have led to a bias in the diagnosis of the medical conditions, particularly in older subjects. However, this potential bias would have been the same across all three compared groups, so that the observed gradient remains meaningful. In addition, the dataset only covered nine major physical comorbidities. Therefore, other physical health comorbidities may well have been evident but not identified in the dataset. Future research should consider additional cardiometabolic conditions, such as hypertension and stroke, which were missing from the evaluated questionnaires. Furthermore, although multimorbidity, defined by two or more physical conditions, has a relevant impact on morbidity, functioning, quality of life, and mortality [12–16], we recognize that specific physical illnesses are different regarding their severity and impact on these outcomes. In addition, due to poor/incomplete data, we were not able to investigate the impact of smoking, substance use, and medication on the relationship between psychosis and multimorbidity. Future research should attempt to assess the degree to which these factors contribute to this relationship. Next, future research should seek to explore the relationship between psychosis and multimorbidity in high-income countries as there may be contextual differences compared with LMICs. For example, as has been reported previously [32], our LMIC sample had a higher prevalence of subclinical psychosis than generally reported figures from high-income countries. Moreover, the sample is based on community dwelling people, whereas other groups of people with psychosis (e.g., inpatients) may have poorer physical health; the study does not account for this at-risk group. Finally, the reliance on self-report and not medical records may mean that our data are actually underestimates. Despite these limitations, the strengths of the study include a large sample size and the multi-national scope, including most regions of the world, but in particular understudied LMICs in Africa, Latin-America, Asia, and Eastern Europe.

## Conclusions

In conclusion, people with psychosis and subclinical psychosis in LMICs had a higher risk for multiple physical conditions. Given that levels of stigma attached to mental disorders may be high in this setting [64], and treatment for both mental disorders and physical health conditions are often suboptimal in LMICs, physical multimorbidity may be associated with a particularly devastating consequence for those with psychosis in this setting. Future studies are needed to assess the impact and cost-effectiveness of global preventive and therapeutic strategies targeting physical multimorbidity in individuals across the full psychotic spectrum.

## Abbreviations

CIs: confidence intervals
LMIC: low- and middle-income countries
OR: odds ratios
SD: standard deviations
WHO: World Health Organization
WHS: World Health Survey.
